# Self-harm in the two years of greatest restrictions during the covid-19 pandemic: a cross-sectional study

**DOI:** 10.1590/0034-7167-2024-0289

**Published:** 2025-01-10

**Authors:** Odilon Vieira Santos, Letícia Aparecida Machado Lisboa da Silva, Meiry Fernanda Pinto Okuno, Paula Hino, José Ramón Martínez-Riera, Hugo Fernandes

**Affiliations:** I Universidade Federal de São Paulo. São Paulo, São Paulo, Brazil; II Universidad de Alicante. Alicante, Spain

**Keywords:** Violence, Suicide, Attempted, Epidemiology, Health Information Systems, Public Health Surveillance, Violencia, Intento de Suicidio, Epidemiología, Sistemas de Información en Salud, Vigilancia en Salud Pública

## Abstract

**Objective:**

to analyze occurrence of self-harm, sociodemographic profile of victims and referrals in the first 24 months of the COVID-19 pandemic in São Paulo.

**Method:**

cross-sectional study carried out by the Notifiable Diseases Information System with data on self-harm in São Paulo. The period outlined was March 2020 to February 2022. R (4.0.2) software and chi-square test were used.

**Results:**

there were 15,946 incidents. Victims were young, white, single, heterosexual women. There was high incidence of people with previous mental disorders more than once and without clear motivation. The method used was poisoning/intoxication. There was a considerable number of referrals to the health network, although not totalitarian.

**Conclusion:**

the years of greater insecurity in relation to the pandemic have given rise to self-harm cases with peculiar characteristics. Agile health policies must be applied in atypical conditions, such as pandemics, especially for adolescents/young people with previous mental disorders.

## RESUMEN

**Objetivo:**

analizar la ocurrencia de violencia autoinfligida, el perfil sociodemográfico de las víctimas y las derivaciones en los primeros 24 meses de la pandemia de COVID-19 en São Paulo.

**Método:**

estudio transversal, realizado por el Sistema de Información de Enfermedades de Declaración Obligatoria con datos sobre violencia autoinfligida en São Paulo. El período reseñado fue de marzo de 2020 a febrero de 2022. Se utilizó el software R (4.0.2) y la prueba de chi cuadrado.

**Resultados:**

ocurrieron 15.946 sucesos. Las víctimas eran mujeres, jóvenes, blancas, solteras y heterosexuales. Hubo una elevada incidencia en personas con trastornos mentales previos más de una vez y sin una motivación clara. El medio utilizado fue envenenamiento/intoxicación. Hubo un número considerable de derivaciones a la red de salud, aunque no total.

**Conclusión:**

los años de mayor inseguridad en relación a la pandemia dieron lugar a casos de violencia autoinfligida con características peculiares. Se deben aplicar políticas de salud ágiles en condiciones atípicas, como pandemias, especialmente para adolescentes/jóvenes con trastornos mentales previos.

## INTRODUCTION

Self-harm, also known as self-injury or self-mutilation, refers to behaviors in which individuals inflict physical harm on themselves as a way to cope with intense emotions, emotional distress, stress, or to express inner pain in some tangible way. One of the most serious forms of self-harm is attempted suicide, in which a person deliberately tries to take their own life^(1)^.

Suicide attempt is an extremely serious behavior that usually results from a complex interaction of several factors, including biological, psychological, social, and environmental factors. People who attempt suicide are often struggling with feelings of hopelessness, loneliness, helplessness, and intense emotional pain. Suicidal ideation can be influenced by mental disorders such as major depression, bipolar disorder, schizophrenia, and others. Past trauma, recent stressful events, financial problems, social isolation, and lack of support can also play a significant role^(1,2)^.

The COVID-19 pandemic has brought with it not only health challenges but also profound impacts on the mental health of people around the world. In this context, the manifestation of self-harm behaviors and suicide attempts has emerged as a significant concern. The complex interplay between the direct and indirect effects of the pandemic has contributed to the worsening of mental health problems and the expression of these self-destructive behaviors in many individuals^(3)^. One of the main reasons for this increase lies in the social isolation imposed by social distancing measures. The lack of in-person social interactions and emotional support that comes with this prolonged isolation have fueled feelings of loneliness, sadness, and hopelessness. These heightened feelings have led people to engage in behaviors such as self-harm as a way to alleviate the emotional pain they are experiencing^(3,4)^.

A US study cites that the economic crisis triggered by the pandemic also contributed to financial stress and uncertainty about the future^(5)^. Job losses, reduced incomes, and widespread financial insecurity have strained people’s coping abilities, making them more prone to mental health problems and self-harm. Uncertainty about the health of themselves and loved ones, and fear of illness and death have also played a role in heightening these behaviors.

Research^(6,7)^ has pointed to an increase in cases of depression, anxiety, panic, mania and dysphoria. Moreover, social problems have become more evident since the beginning of the pandemic, especially in times of restrictions on commercial activities imposed as a complementary measure to reduce the movement of people in epidemiological moments of increasing number of cases and risk of collapse in the public health system. Thus, when the factors listed are combined, many people may develop conditions that affect them so intimately and strongly that they can trigger behaviors of self-harm and/or attempted suicide.

Violence has been a public health concern for years, and has been on the list of notifiable injuries since 2009, including self-harm and attempted suicide. The latter is immediately notifiable, meaning a person notifying the incident must inform the epidemiological surveillance agencies immediately after providing initial care to victims so that control measures and specialized care can be implemented quickly, preventing relapses in suicidal behavior or worsening of the psychological condition^(8)^.

A systematic review that aimed to analyze the incidence of suicide attempts and their trends during the COVID-19 pandemic pointed out the low number of epidemiological studies on the subject, which detailed occurrence characteristics, causes and measures adopted by the health sector in assisting victims^(9)^. Thus, the following questions arose among the authors: what are the sociodemographic characteristics of victims of self-harm during the COVID-19 pandemic, the conditions under which it occurred, and what referrals were made by healthcare professionals? It is estimated that the results can contribute to implementing health policies for disease prevention and control in atypical situations, such as pandemics and catastrophes.

## OBJECTIVE

To analyze occurrence of self-harm, sociodemographic profile of victims and referrals in the first 24 months of the COVID-19 pandemic in São Paulo.

## METHOD

### Ethical aspects

The study was conducted in accordance with established ethical principles for research. Since the data were collected from a public domain and widely accessible database, it was not necessary to submit the project to a Research Ethics Committee, following international standards and Resolution 466/12 of the Brazilian National Health Council.

### Study design

This study has a descriptive and exploratory approach, with a cross-sectional design^(10)^, following the STrengthening the Reporting of OBservational studies in Epidemiology (STROBE) guidelines^(11)^.

### Data collection

The data were collected in the city of São Paulo, SP. Information was obtained through the Notification Forms for Suspected or Confirmed Cases of Interpersonal and Self-harm from the Notifiable Diseases Information System (SINAN - *Sistema de Informação de Agravos de Notificação*). The information was recorded and stored in SINAN by several notifying services, including epidemiological surveillance, hospitals and outpatient clinics. After the collection stage, the information was made available by the Municipal Health Department of São Paulo in the TabNet tabulation software, developed by the Brazilian Health System Department of Information Technology and accessible online.

São Paulo was chosen due to its prominence as the most populous city in Brazil, combined with its remarkable social and cultural diversity. Additionally, notification data in the city was easier to access for public consultation through TabNet, in contrast to the national system. While information for the capital of São Paulo was available until 2022, the national system only offered tabulated data until 2020.

### Variables studied

The study analyzed victims’ variables, including age range, gender, race/skin color, pregnancy, education, health coordinator of victims’ residence, marital status, sexual orientation, gender identity, presence of disorder or disability and its type. As for the occurrences, the following were analyzed: month, location, whether it occurred more than once, motivation, means of aggression, suspicion of alcohol use and referrals made by professionals, after treating victims.

### Eligibility criteria

Notifications of self-harm made in both public and private health facilities located in São Paulo, SP, were used. Cases of interpersonal violence and duplicate reports were excluded. Due to the uniformity of the notification form for the spectrum of suspected and confirmed cases of interpersonal violence, a segregation among these categories proved unfeasible. Furthermore, it should be noted that the TabNet program does not distinguish between confirmed and suspected cases. In this context, it was decided to include all occurrences of self-harm, whether they were considered suspected or already confirmed at the time of notification.

### Analysis period

Data collection took place from March 2020 to February 2022, totaling 24 months. This interval was chosen because the World Health Organization (WHO) declared the pandemic in March 2020, and social distancing measures began to be implemented in São Paulo days later. Data collection was carried out between December 2022 and February 2023. Microsoft Excel 365^®^ was used to analyze the data.

### Data treatment and analysis

Univariate statistical analysis was conducted using R software version 4.0.2. Given that the data set is composed of categorical variables, a descriptive analysis was performed by counting absolute and percentage frequencies. To examine the associations between violence and other variables, such as motivation for violence, means of aggression, location, aggressor gender, referral, age group, race, education, marital status, and disability, the chi-square test was used. The significance level adopted was 5% (α = 0.05).

## Results

During the period outlined, there were 15,946 notifications of self-harm in São Paulo, SP. Statistically significant values were found in most sociodemographic variables with the exception of “pregnancy” (p=0.460) and “physical disability” (p=0.086), as shown in Table 1.

**Table 1:** Notifications of self-harm according to sociodemographic characteristics of victims. São Paulo, SP, Brazil, 2023

Variable	n	%	*p* value^*^
Age group (years)			<0.001
0 - 4	177	1.1	
5 - 9	68	0.4	
10 - 14	1605	10	
15 - 19	3677	23	
20 - 24	2996	18.8	
25 - 29	1963	12.3	
30 - 34	1444	9	
35 - 39	1230	7.8	
40 - 44	994	6.2	
45 - 49	661	4.2	
50 - 54	453	2.8	
55 - 59	256	1.7	
60 - 64	179	1.1	
65 - 69	112	0.7	
70 - 74	59	0.4	
75 and over	73	0.5	
Sex			<0.001
Female	11141	69.9	
Male	4804	30.1	
Race/color			0.037
Ignored/blank	1116	7	
White	7702	48.3	
Black	1479	9.3	
Yellow	121	0.7	
Brown	5481	34.4	
Indigenous	48	0.3	
Gestation			0.460
Ignored/blank	2339	14.7	
Yes	271	1.7	
No	7242	45.4	
Not applicable	6095	38.3	
1^st^ trimester	99	0.6	
2^nd^ trimester	97	0.6	
3^rd^ trimester	51	0.3	
Gestational age unknown	24	0.1	
Education			0.009
Ignored/blank	6132	38.5	
Illiterate	53	0.3	
Incomplete 1^st^ to 4^th^ grade of elementary school	296	1.9	
Incomplete 5^th^ to 8^th^ grade of elementary school	231	1.4	
Complete elementary school	1723	10.8	
Incomplete high school	861	5.4	
Complete high school	2230	14.0	
Incomplete higher education	3027	19.0	
Complete higher education	639	4.0	
Not applicable	578	3.6	
Residential health coordination (zone)			<0.001
West	973	6.1	
East	2504	15.7	
North	3095	19.4	
Southeast	2776	17.4	
South	4713	29.6	
Center	381	2.4	
Blank	1523	9.6	
Not provided	8	0.1	
Marital status			<0.001
Blank	204	1.3	
Single	7684	48.2	
Married/consensual union	2609	16.4	
Widowed	117	0.7	
Separated	575	3.6	
Not applicable	1104	6.9	
Ignored	3680	23.1	
Sexual orientation			<0.001
Heterosexual	8006	50.2	
Homosexual	597	3.7	
Bisexual	262	1.6	
Not applicable	1111	7.0	
Ignored	5997	37.6	
Gender identity			<0.001
Transvestite	35	0.2	
Transsexual woman	182	1.1	
Transsexual man	135	0.8	
Not applicable	9205	57.7	
Ignored	6416	40.2	
Presence of disability or disorder			<0.001
Blank	62	0.4	
Yes	4644	29.1	
No	7862	49.3	
Ignored	3405	21.4	
Physical disability			0.086
Blank	162	1.0	
Yes	100	0.6	
No	4311	27.0	
Not applicable	11264	70.6	
Ignored	136	0.9	
Intellectual disability			<0.001
Blank	158	1.0	
Yes	307	1.9	
No	4109	25.8	
Not applicable	11264	70.6	
Ignored	135	0.8	
Mental disorder			<0.001
Blank	124	0.8	
Yes	2036	12.8	
No	2454	15.4	
Not applicable	11264	70.6	
Ignored	95	0.6	
Behavioral disorder			<0.001
Blank	121	0.8	
Yes	2260	14.2	
No	2242	14.1	
Not applicable	11264	70.6	
Ignored	86	0.5	

* *Chi-square test.*

There was a higher frequency of notifications of self-harm suffered by people aged between 15 and 19 years (23%; n=3,677), followed by people aged between 20 and 24 years (18.8%; n=2,996), female (69.9%; n=11,151), white (48.3%; n=7,702), single (48.2%; n=7,684), heterosexual (50.2%; n=8,006) and residents in the southern region of the health coordination (29.6%; n=4,713). Concerning education, there was a high number of records classified as “unknown/blank” (38.5%; n=6,132).

As for the presence of disability or disorder, the majority did not have these conditions (49.3%; n=7,862). However, in affirmative cases, behavioral disorders were the most prevalent (14.2%; n=2,260), followed by mental disorders (12.8%; n=2,036).

Table 2 presents the main characteristics regarding the occurrence of self-harm, such as the location of the occurrence, whether it occurred other times, motivation and means of aggression.

**Table 2:** Notifications of self-harm according to occurrence characteristics. São Paulo, SP, Brazil, 2023

Variable	n	%	*p* value^*^
Place of occurrence			0.033
Blank	4	0.0	
Residence	12197	76.5	
Collective housing	108	0.7	
School	53	0.3	
Sports venue	5	0.0	
Bar or similar	20	0.1	
Public road	782	4.9	
Commerce/services	122	0.8	
Industry/construction	5	0.0	
Others	429	2.7	
Ignored	2255	14.1	
Occurred other times			
Blank	192	1.2	0.002
Yes	7182	45.0	
No	4816	30.2	
Ignored	3790	23.8	
Motivation			
Sexism	115	0.7	<0.001
Homophobia/transphobia	77	0.5	
Racism	10	0.1	
Religious intolerance	5	0.0	
Xenophobia	5	0.0	
Generational conflict	911	5.7	
Homelessness	43	0.3	
Disability	117	0.7	
Others	5405	33.9	
Not applicable	3105	19.5	
Ignored	6187	38.8	
Means of aggression			
Bodily force	278	1.7	<0.001
Hanging	578	3.6	
Blunt object	194	1.2	
Sharp object	3408	21.4	
Hot substance or object	107	0.7	
Poisoning or intoxication	9733	61.0	
Firearm	68	0.4	
Threat	309	1.9	
Other means	1626	10.2	
Suspected alcohol use			
Blank	111	0.7	0.004
Yes	2360	14.8	
No	8665	54.3	
Ignored	4844	30.4	

* *Chi-square test.*

Table 2 shows that the most common location of self-harm was the victim’s home (76.5%; n=12,197) and that the aggression occurred more frequently than once (45%; n=7,182). The main motivation for self-harm is not clear, given that most of the notifications had the “ignored” field as the most filled out (38.8%; n=6,187), followed by “others” (33.9%; n=5,405).

The most common means of aggression was poisoning or intoxication (61%; n= 9,733) and was not associated with alcohol consumption in most cases (54%; n= 8,665).

Table 3 summarizes referrals made by professionals after assisting victims.

**Table 3:** Referrals made by professionals after assisting victims of self-harm. São Paulo, SP, Brazil, 2023

Referrals	n	%	*p* value^*^
Health Care Network	13558	76.5	<0.001
Social Assistance Network	910	5.1	
Education Network	55	0.3	
Women’s Care Network	519	2.9	
Tutelary Council	2276	12.8	
Elderly Council	28	0.2	
Human Rights Reference Center	9	0.1	
Public Prosecutor’s Office	13	0.1	
Child and Adolescent Protection Police Station	33	0.2	
Women’s Care Police Station	46	0.3	
Other police stations	221	1.2	
Child and Youth Justice	42	0.2	
Public Defender’s Office	13	0.1	
Total	17723	100	

* *Chi-square test.*

It is noted that professionals referred victims of self-harm mainly to services that make up the Health Care Network (76.5%; n=13,558). It is noteworthy that, in this field, professionals could indicate more than one type of referral. Thus, the total value (n=17,723) is higher than the number of cases attended to in the outlined period (n=15,946).

## DISCUSSION

Violence has profound consequences for the physical and mental health of those who experience it, impacting their psychosocial development and well-being. This study found that young people aged 15 to 24 were the main victims of self-harm and attempted suicide during the pandemic. According to the WHO^(12)^, suicide is the fourth leading cause of death among young people in this age group, and the suicide rate among adolescents has increased in many countries in recent decades. Mortality among adolescents and young adults compromises the future and points to the neglect of governments and societies in ensuring a full and quality life for this age group.

A Brazilian study, carried out at the beginning of the COVID-19 pandemic, with care records of 130 patients treated after suicidal ideation or attempted suicide in the psychiatric emergency room of a mental healthcare service, identified that there was a high incidence of suicidal behavior among adolescents aged 13 and over^(13)^. Other research indicates that factors associated with self-harm by adolescents in the pandemic context were associated with environmental, emotional, genetic and physiological aspects, in addition to other conditions such as anxiety and abuse of illicit substances^(1,5)^.

In terms of gender, men and women can be victims of self-harm at different times in their life cycle. However, women suffer more frequently from mental disorders and socio-environmental conditions that make them more vulnerable to attempted suicide. A Canadian study on the subject during the COVID-19 pandemic found that women had a higher frequency of suicidal ideation and that aspects related to social isolation, increased unemployment, lack of support network, insecurity about the near future and increased domestic demands were factors driving this behavior^(14)^.

Vulnerability is even greater when considering the intersection between gender, poverty and race/color, with brown and black women having a higher incidence of suicidal ideation. However, this aspect was not evidenced in this study, given that most victims self-declared as white. It is worth noting that self-declaration of skin color in Brazil is a sensitive issue due to the structural racism found in the country, causing black people often not to recognize themselves as such^(15)^. The condition of poverty was not investigated in this study, but the high occurrence of the problem in the southern region of the municipality, where large areas of poverty and intense social inequality are present, may suggest such an intersection.

Regarding characteristics related to marital status, there is evidence in the literature that describes the prevalence of suicide attempts and suicide in single people. Data from a quantitative retrospective study that analyzed the epidemiological profile of suicide attempts and deaths in Brazil between 2009 and 2018 revealed that the marital status of most victims in all regions of the Brazilian territory was single^(16)^. Likewise, in analyses by researchers at the *Universidade de Brasília*, higher percentages of deaths by suicide in the Federal District were found among single, widowed and legally separated individuals compared to individuals in a stable union^(17)^.

Another result found refers to suicidal behavior and practice in individuals who declare themselves heterosexual. The data found in the present study differ from a national study that points to greater vulnerability and occurrence in non-heterosexual individuals, justified by the influence of discriminatory factors experienced by homosexuals and bisexuals^(18)^. International literature also points to a higher prevalence of suicidal behavior in non-heterosexual people^(19)^. Thus, the findings of this research reveal that, during the COVID-19 pandemic, discrimination associated with homosexuality or bisexuality was not a primary motivator for self-harm.

A high frequency of “other”, “blank” or “ignored” results in fields such as victims’ education level may indicate failures in the training of notifiers, requiring increased awareness that compulsory notification is an important tool not only for monitoring and investigating cases of violence, but also as a fundamental instrument for decision-making in health, such as the implementation of public policies^(20)^, a fact found in this research.

People with previous behavioral and mental disorders had a higher frequency of self-violence. These groups include antisocial personality disorders, borderline personality disorder, histrionic personality disorder, depression, anxiety, bipolar affective disorder, schizophrenia, among other conditions. International researchers cite a close relationship between mental and behavioral disorders and suicidal ideation during the pandemic, especially among adolescents and young adults^(21)^. However, it is not yet clear whether in all cases such disorders were previously existing, latent or developed due to the conditions of social isolation and fear caused by the COVID-19 pandemic.

The occurrence of self-harm and suicide attempts were more common in victims’ homes, possibly due to pandemic restrictions and also because it is a type of intimate violence that does not require public spaces for its occurrence. A relevant finding was that the aggravation occurred more than once in approximately 45% of notifications. Repeated self-harm expresses a sign of the severity of victims’ mental status, requiring rapid and assertive interventions, given the high probability of suicide success due to the improvement of the practice that previously failed^(21,22)^. Since it has variable causes, their motivation is not always clear or can be expressed by victims clearly, causing healthcare professionals who assisted them to not identify appropriate fields to fill in the compulsory notification form^(8,20)^.

As for the means of aggression, poisoning or intoxication was the most frequent. However, the literature shows a variation in the methods most used both nationally and internationally^(21-23)^. Among the methods frequently used to commit suicide, an epidemiological literature review showed that there was a predominance of self-inflicted deaths by hanging, accounting for 47% of cases, followed by firearms (19%) and poisoning (14%)^(23)^. In the international scenario, the same study pointed out the use of pesticide and medication poisoning in Asian countries, such as China and Sri Lanka. It is worth noting that there are factors linked to the socioeconomic and cultural context in which a person is inserted that directly influence the choice of method. Therefore, the most used forms can vary greatly.

Similarly, Brazilian researchers described in a cross-sectional study cases of self-harm and exogenous poisoning that occurred in Rio Grande do Sul between 2013 and 2017^(24)^. In accordance with the findings of the analysis, it was found in the sample that most cases of self-harm were caused by poisoning or exogenous intoxication, accounting 39.4% of the notifications in the state of Rio Grande do Sul. Subsequently, came deaths caused by hanging, use of sharp objects and firearms, respectively.

Finally, the high number of referrals to the Health Care Network is justified by the need for specialized mental healthcare required in these situations, in addition to signaling alignment with the recommendations of the line of care for people in situations of violence in the city of São Paulo. However, it is noted that not all cases were referred to healthcare services specialized in mental health, such as psychiatrists and/or psychologists, as recommended by the aforementioned line of care^(25)^. Failure to refer victims quickly to specialized healthcare professionals can put victims’ integrity at risk and increase the chances of relapses^(22,25)^.

### Study limitations

One of the main limitations of this study is the regional nature of the facts, given that, as it is the largest metropolis in Latin America, the profile of victims and the characteristics of violence perpetrated against them may be different from smaller cities. Furthermore, in this city, all Basic Health Units have Violence Prevention Centers, which are composed of health and/or social assistance professionals trained in the approach to care for victims of violence, whether interpersonal or self-inflicted. In regions where there are no such centers or specialized lines of care, there may be a deficit in notifying and care for victims.

### Contributions to nursing and science

Despite the limitations exposed, the study contributes to the advancement of science, as it explores the novelty of the topic and presents a sociodemographic profile and the main characteristics of self-harm in an atypical situation, such as the COVID-19 pandemic, and raises the need for reflections on future strategies to reduce this problem in similar situations, including prevention in highly vulnerable populations, such as adolescent and young adult women.

## CONCLUSION

Self-harm increased during the COVID-19 pandemic, with the main victims being adolescent and young adult women, white, single, heterosexual, living in the region with the greatest social inequality in the city. Those with mental or behavioral disorders had a higher frequency of the attack, and it is necessary to seek treatment and assistance that takes into account the vulnerability to self-harm. Unfortunately, violence occurred more than once, increasing the chances of a successful suicide attempt and highlighting potential flaws in problem-solving.

Poisoning or intoxication was the most common method used by victims, whose motivation for the occurrence was classified as unknown. The Health Care Network was the main referral made after care and indicates the following of the recommendations of the line of care for people in situations of violence.

The COVID-19 pandemic has caused innumerable damage to society, with likely mental and behavioral consequences. This research has shed light on the issue of self-harm in atypical situations, such as the pandemic. However, it is important to highlight that the fields of mental health and health surveillance continue to develop in search of risk reduction strategies, such as more detailed and accurate mandatory notification, and by approaching this problem in a holistic way that takes into account the multiple factors that influence it.

## Supplementary Material

0034-7167-reben-77-s2-e20240289-supl01

## Data Availability

https://osf.io/u7ek8/
