# Supplementary material for: Self-harm in the two years of greatest restrictions during the covid-19 pandemic: a cross-sectional study
Source: Rev Bras Enferm. 2025 Jan 10;77(Suppl 2):e20240289. doi: 10.1590/0034-7167-2024-0289 (PMC11726816; doi:10.1590/0034-7167-2024-0289)
Supplement: 0034-7167-reben-77-s2-e20240289-supl01 [file 0034-7167-reben-77-s2-e20240289-supl01.pdf]

INSTRUMENTO DE COLETA DE DADOS						
2020		2021		2022		TOTAL 24 MESES
VARIÁVEL	Nº	VARIÁVEL	Nº	VARIÁVEL	Nº	
**FAIXA ETÁRIA**						
0 - 4 ANOS	76	0 - 4 ANOS	92	0 - 4 ANOS	9	177
5 - 9 ANOS	32	5 - 9 ANOS	31	5 - 9 ANOS	5	68
10 - 14 ANOS	525	10 - 14 ANOS	916	10 - 14 ANOS	164	1.605
15 - 19 ANOS	1.427	15 - 19 ANOS	1.926	15 - 19 ANOS	324	**3.677**
20 - 24 ANOS	1.147	20 - 24 ANOS	1.616	20 - 24 ANOS	233	2.996
25 - 29 ANOS	694	25 - 29 ANOS	1.114	25 - 29 ANOS	155	1.963
30 - 34 ANOS	597	30 - 34 ANOS	727	30 - 34 ANOS	120	1.444
35 - 39 ANOS	517	35 - 39 ANOS	615	35 - 39 ANOS	98	1.230
40 - 44 ANOS	406	40 - 44 ANOS	499	40 - 44 ANOS	89	994
45 - 49 ANOS	261	45 - 49 ANOS	341	45 - 49 ANOS	59	661
50 - 54 ANOS	167	50 - 54 ANOS	248	50 - 54 ANOS	38	453
55 - 59 ANOS	106	55 - 59 ANOS	136	55 - 59 ANOS	14	256
60 - 64 ANOS	78	60 - 64 ANOS	85	60 - 64 ANOS	16	179
65 - 69 ANOS	45	65 - 69 ANOS	60	65 - 69 ANOS	7	112
70 - 74 ANOS	18	70 - 74 ANOS	34	70 - 74 ANOS	7	59
75 E MAIS	40	75 E MAIS	31	75 E MAIS	2	73
**SEXO**						
FEMININO	4.233	FEMININO	5.968	FEMININO	940	**11.141**
MASCULINO	1.902	MASCULINO	2.503	MASCULINO	399	4.804
**RAÇA**						
IGN/BRANCO	451	IGN/BRANCO	593	IGN/BRANCO	72	1.116
BRANCA	2.926	BRANCA	4.132	BRANCA	644	**7.702**
PRETA	581	PRETA	760	PRETA	138	1.479
AMARELA	39	AMARELA	71	AMARELA	11	121
PARDA	2.125	PARDA	2.888	PARDA	468	5.481
INDÍGENA	14	INDÍGENA	27	INDÍGENA	7	78
**GESTANTE**						
IGN/BRANCO	961	IGN/BRANCO	1.229	IGN/BRANCO	149	2.339
SIM	111	SIM	134	SIM	26	271
NÃO	2.677	NÃO	3.921	NÃO	644	**7.242**
NÃO SE APLICA	2.387	NÃO SE APLICA	3.187	NÃO SE APLICA	521	6.095
1º TRIMESTRE	39	1º TRIMESTRE	51	1º TRIMESTRE	9	**99**
2º TRIMESTRE	43	2º TRIMESTRE	44	2º TRIMESTRE	10	97
3º TRIMESTRE	19	3º TRIMESTRE	28	3º TRIMESTRE	4	51
IG IGNORADA	10	IG IGNORADA	11	IG IGNORADA	3	24
**ESCOLARIDADE**						
IGN/BRANCO	2.348	IGN/BRANCO	3.300	IGN/BRANCO	484	6.132
ANALFABETO	21	ANALFABETO	27	ANALFABETO	5	53
INCOMPLETA EF	139	INCOMPLETA EF	134	INCOMPLETA EF	23	296
COMPLETA EF	103	COMPLETA EF	114	COMPLETA EF	14	231
INCOMPLETA EF	643	INCOMPLETA EF	947	INCOMPLETA EF	133	1.723
EF COMPLETO	327	EF COMPLETO	439	EF COMPLETO	95	861
EM INCOMPLETO	856	EM INCOMPLETO	1.184	INCOMPLETO	190	2.230
EM COMPLETO	1.149	EM COMPLETO	1.594	EM COMPLETO	284	**3.027**
ES INCOMPLETA	253	ES INCOMPLETA	332	ES INCOMPLETA	54	639

ES COMPLETA	205	ES COMPLETA	324	ES COMPLETA	49	578
NÃO SE APLICA	92	NÃO SE APLICA	100	NÃO SE APLICA	11	203
**COORDENADORIA DE SAÚDE (RESIDÊNCIA)**						
OESTE	322	OESTE	579	OESTE	72	973
LESTE	992	LESTE	1.316	LESTE	196	2.504
NORTE	1.225	NORTE	1.603	NORTE	267	3.095
SUDESTE	976	SUDESTE	1.546	SUDESTE	254	2.776
SUL	1.939	SUL	2.383	SUL	391	**4.713**
CENTRO	157	CENTRO	200	CENTRO	24	381
EM BRANCO	525	EM BRANCO	863	EM BRANCO	135	1.523
		CLASSIFICADO	5	CLASSIFICADO	3	8
**SITUAÇÃO CONJUGAL**						
EM BRANCO	58	EM BRANCO	126	EM BRANCO	20	204
SOLTEIRO	2.937	SOLTEIRO	4.064	SOLTEIRO	683	**7.684**
CONSENSUAL	1.044	CONSENSUAL	1.368	CONSENSUAL	197	2.609
VIÚVO	54	VIÚVO	57	VIÚVO	6	117
SEPARADO	223	SEPARADO	304	SEPARADO	48	575
NÃO SE APLICA	361	NÃO SE APLICA	629	NÃO SE APLICA	114	1.104
IGNORADO	1.459	IGNORADO	1.947	IGNORADO	274	3.680
**ORIENTAÇÃO SEXUAL**						
HETEROSSEXUAL	3.188	HETEROSSEXUAL	4.137	HETEROSSEXUAL	681	**8.006**
HOMOSSEXUAL	233	HOMOSSEXUAL	319	HOMOSSEXUAL	45	597
BISSEXUAL	58	BISSEXUAL	174	BISSEXUAL	30	262
NÃO SE APLICA	420	NÃO SE APLICA	602	NÃO SE APLICA	89	1.111
IGNORADO	2.237	IGNORADO	3.263	IGNORADO	497	5.997
**IDENTIDADE DE GÊNERO**						
TRAVESTI	14	TRAVESTI	20	TRAVESTI	1	35
TRAVESTI	65	MULHER TRAVESTI	107	TRAVESTI	10	182
TRAVESTI	29	HOMEM TRAVESTI	82	TRAVESTI	24	135
NÃO SE APLICA	3.485	NÃO SE APLICA	4.883	NÃO SE APLICA	837	**9.205**
IGNORADO	2.543	IGNORADO	3.403	IGNORADO	470	6.416
**POSSUI DEFICIÊNCIA/TRANSTORNO MENTAL**						
EM BRANCO	17	EM BRANCO	37	EM BRANCO	8	62
SIM	1.761	SIM	2.507	SIM	376	4.644
NÃO	2.863	NÃO	4.300	NÃO	699	**7.862**
IGNORADO	1.495	IGNORADO	1.651	IGNORADO	259	3.405
**TIPOS DE DEFICIÊNCIA**						
**TIPO DE DEFICIÊNCIA: DEFICIÊNCIA FÍSICA**						
EM BRANCO	53	EM BRANCO	77	EM BRANCO	32	162
SIM	49	SIM	45	SIM	6	100
NÃO	1.603	NÃO	2.366	NÃO	342	4.311
NÃO SE APLICA	4.357	NÃO SE APLICA	5.949	NÃO SE APLICA	958	**11.264**
IGNORADO	74	IGNORADO	58	IGNORADO	4	136
**TIPO DE DEFICIÊNCIA: DEFICIÊNCIA INTELECTUAL**						
EM BRANCO	50	EM BRANCO	77	EM BRANCO	31	158
SIM	158	SIM	120	SIM	29	307
NÃO	1.499	NÃO	2.293	NÃO	317	4.109
NÃO SE APLICA	4.357	NÃO SE APLICA	5.949	NÃO SE APLICA	958	**11.274**
IGNORADO	72	IGNORADO	56	IGNORADO	7	135
**TIPO DE DEFICIÊNCIA: DEFICIÊNCIA VISUAL**						

EM BRANCO	51	EM BRANCO	76	EM BRANCO	32	159
SIM	12	SIM	24	SIM	2	38
NÃO	1.640	NÃO	2.388	NÃO	345	4.373
NÃO SE APLICA	4.357	NÃO SE APLICA	5.949	NÃO SE APLICA	958	**11.264**
IGNORADO	76	IGNORADO	58	IGNORADO	5	139
**TIPO DE DEFICIÊNCIA: DEFICIÊNCIA AUDITIVA**						
EM BRANCO	55	EM BRANCO	77	EM BRANCO	34	166
SIM	13	SIM	12	SIM	4	29
NÃO	1.635	NÃO	2.398	NÃO	341	4.374
NÃO SE APLICA	4.357	NÃO SE APLICA	5.949	NÃO SE APLICA	958	**11.264**
IGNORADO	76	IGNORADO	59	IGNORADO	5	137
**TIPO DE DEFICIÊNCIA: TRANSTORNO MENTAL**						
EM BRANCO	40	EM BRANCO	60	EM BRANCO	24	124
SIM	858	SIM	1.035	SIM	143	2.036
NÃO	833	NÃO	1.409	NÃO	212	2.454
NÃO SE APLICA	4.357	NÃO SE APLICA	5.949	NÃO SE APLICA	958	**11.264**
IGNORADO	48	IGNORADO	42	IGNORADO	5	95
**TIPO DE DEFICIÊNCIA: TRANSTORNO DE COMPORTAMENTO**						
EM BRANCO	41	EM BRANCO	66	EM BRANCO	14	121
SIM	762	SIM	1.282	SIM	216	2.260
NÃO	935	NÃO	1.158	NÃO	149	2.242
NÃO SE APLICA	4.357	NÃO SE APLICA	5.949	NÃO SE APLICA	958	**11.264**
IGNORADO	41	IGNORADO	40	IGNORADO	5	86
**MÊS DA OCORRÊNCIA**						
JANEIRO	X	JANEIRO	711	JANEIRO	592	1.303
FEVEREIRO	X	FEVEREIRO	705	FEVEREIRO	640	1.345
MARÇO	692	MARÇO	617	MARÇO	X	1.309
ABRIL	407	ABRIL	649	ABRIL	X	1.056
MAIO	385	MAIO	715	MAIO	X	1.100
JUNHO	514	JUNHO	666	JUNHO	X	1.180
JULHO	596	JULHO	626	JULHO	X	1.222
AGOSTO	658	AGOSTO	749	AGOSTO	X	1.407
SETEMBRO	771	SETEMBRO	834	SETEMBRO	X	**1.605**
OUTUBRO	754	OUTUBRO	834	OUTUBRO	X	1.588
NOVEMBRO	723	NOVEMBRO	776	NOVEMBRO	X	1.499
DEZEMBRO	622	DEZEMBRO	620	DEZEMBRO	X	1.242
**LOCAL DA OCORRÊNCIA**						
EM BRANCO	3	EM BRANCO	1	EM BRANCO	X	4
RESIDÊNCIA	4.638	RESIDÊNCIA	6.517	RESIDÊNCIA	1.042	**12.197**
COLETIVA	44	COLETIVA	55	COLETIVA	9	108
ESCOLA	16	ESCOLA	27	ESCOLA	10	53
PRÁTICA	X	ESPORTIVA	2	PRÁTICA	3	5
BAR OU SIMILAR	6	BAR OU SIMILAR	12	BAR OU SIMILAR	2	20
VIA PÚBLICA	325	VIA PÚBLICA	390	VIA PÚBLICA	67	782
ÇOS	56	OS	57	IÇOS	9	122
STRUÇÃO	3	TRUÇÃO	2	NSTRUÇÃO	X	5
OUTROS	174	OUTROS	223	OUTROS	32	429
IGNORADO	871	IGNORADO	1.216	IGNORADO	168	2.255
**OCORREU OUTRAS VEZES**						
EM BRANCO	72	EM BRANCO	98	EM BRANCO	22	192

SIM	2.755	SIM	3.791	SIM	636	**7.362**
NÃO	1.802	NÃO	2.599	NÃO	415	4.816
IGNORADO	1.507	IGNORADO	2.014	IGNORADO	269	3.790
**MOTIVAÇÃO DA VIOLÊNCIA**						
SEXISMO	51	SEXISMO	51	SEXISMO	13	115
NSFOBIA	31	NSFOBIA	43	ANSFOBIA	3	77
RACISMO	3	RACISMO	5	RACISMO	2	10
RELIGIOSA	2	RELIGIOSA	3	RELIGIOSA	X	5
XENOFOBIA	3	XENOFOBIA	1	XENOFOBIA	1	5
GERACIONAL	380	GERACIONAL	454	GERACIONAL	77	911
RUA	15	SITUAÇÃO DE RUA	26	RUA	2	29
DEFICIÊNCIA	44	DEFICIÊNCIA	70	DEFICIÊNCIA	3	117
OUTROS	1.907	OUTROS	3.033	OUTROS	465	5.405
NÃO SE APLICA	1.014	NÃO SE APLICA	1.822	NÃO SE APLICA	269	3.105
IGNORADO	2.686	IGNORADO	2.994	IGNORADO	507	**6.187**
**MEIO DE AGRESSÃO**						
**FORÇA CORPORAL/ESPANCAMENTO**						
EM BRANCO	321	EM BRANCO	377	EM BRANCO	77	775
SIM	109	SIM	145	SIM	24	278
NÃO	5.519	NÃO	7.845	NÃO	1.235	**14.599**
IGNORADO	187	IGNORADO	135	IGNORADO	6	328
**ENFORCAMENTO**						
EM BRANCO	316	EM BRANCO	371	EM BRANCO	75	762
SIM	233	SIM	303	SIM	42	578
NÃO	5.398	NÃO	7.697	NÃO	1.219	**14.314**
IGNORADO	189	IGNORADO	131	IGNORADO	6	326
**OBJETO CONTUNDENTE**						
EM BRANCO	325	EM BRANCO	389	EM BRANCO	76	790
SIM	69	SIM	109	SIM	16	194
NÃO	5.550	NÃO	7.871	NÃO	1.244	**14.665**
IGNORADO	192	IGNORADO	133	IGNORADO	6	331
**OBJETO PÉRFURO-CORTANTE**						
EM BRANCO	253	EM BRANCO	303	EM BRANCO	61	617
SIM	1.265	SIM	1.824	SIM	319	3.408
NÃO	4.448	NÃO	6.243	NÃO	956	**11.647**
IGNORADO	170	IGNORADO	132	IGNORADO	6	308
**SUBSTÂNCIA/OBJETO QUENTE**						
EM BRANCO	324	EM BRANCO	386	EM BRANCO	76	786
SIM	51	SIM	53	SIM	3	107
NÃO	5.568	NÃO	7.932	NÃO	1.257	**14.757**
IGNORADO	193	IGNORADO	131	IGNORADO	6	330
**ENVENENAMENTO**						
EM BRANCO	151	EM BRANCO	182	EM BRANCO	29	362
SIM	3.611	SIM	5.280	SIM	842	**9.733**
NÃO	2.249	NÃO	2.941	NÃO	468	5.658
IGNORADO	125	IGNORADO	99	IGNORADO	3	227
**ARMA DE FOGO**						
EM BRANCO	331	EM BRANCO	391	EM BRANCO	78	800
SIM	33	SIM	27	SIM	8	68
NÃO	5.578	NÃO	7.951	NÃO	1.251	**14.780**

IGNORADO	194	IGNORADO	133	IGNORADO	5	332
**AMEAÇA**						
EM BRANCO	319	EM BRANCO	372	EM BRANCO	75	766
SIM	141	SIM	149	SIM	19	309
NÃO	5.494	NÃO	7.848	NÃO	1.243	**14.585**
IGNORADO	182	IGNORADO	133	IGNORADO	5	320
**OUTRO MEIO**						
EM BRANCO	338	EM BRANCO	379	EM BRANCO	83	800
SIM	698	SIM	802	SIM	126	1.626
NÃO	4.868	NÃO	7.145	NÃO	1.121	**13.134**
IGNORADO	232	IGNORADO	176	IGNORADO	12	420
**SUSPEITA DE USO DE ÁLCOOL**						
EM BRANCO	44	EM BRANCO	55	EM BRANCO	12	111
SIM	979	SIM	1.189	SIM	192	2.360
NÃO	3.039	NÃO	4.865	NÃO	761	**8.665**
IGNORADO	2.074	IGNORADO	2.393	IGNORADO	377	4.844
**ENCAMINHAMENTOS REALIZADOS**						
**ENCAMINHAMENTO: REDE DE ATENÇÃO À SAÚDE**						
EM BRANCO	1	EM BRANCO	2	EM BRANCO	X	3
SIM	4.949	SIM	7.432	SIM	1.177	13.558
NÃO	770	NÃO	875	NÃO	155	1.800
IGNORADO	416	IGNORADO	193	IGNORADO	10	619
**ENCAMINHAMENTO: REDE DA ASSISTÊNCIA SOCIAL**						
EM BRANCO	38	EM BRANCO	61	EM BRANCO	10	119
SIM	384	SIM	468	SIM	58	910
NÃO	5.233	NÃO	7.642	NÃO	1.246	**14.121**
IGNORADO	481	IGNORADO	331	IGNORADO	28	840
**ENCAMINHAMENTO: REDE DA EDUCAÇÃO**						
EM BRANCO	42	EM BRANCO	66	EM BRANCO	11	119
SIM	11	SIM	40	SIM	4	55
NÃO	5.612	NÃO	8.064	NÃO	1.299	**14.975**
IGNORADO	471	IGNORADO	332	IGNORADO	28	831
**ENCAMINHAMENTO: REDE DE ATENDIMENTO À MULHER**						
EM BRANCO	45	EM BRANCO	67	EM BRANCO	13	125
SIM	183	SIM	292	SIM	44	519
NÃO	5.433	NÃO	7.818	NÃO	1.257	**14.508**
IGNORADO	475	IGNORADO	325	IGNORADO	28	828
**ENCAMINHAMENTO: CONSELHO TUTELAR**						
EM BRANCO	47	EM BRANCO	67	EM BRANCO	13	127
SIM	821	SIM	1.264	SIM	191	2.276
NÃO	4.808	NÃO	6.852	NÃO	1.110	**12.770**
IGNORADO	460	IGNORADO	319	IGNORADO	28	807
**ENCAMINHAMENTO: CONSELHO DO IDOSO**						
EM BRANCO	48	EM BRANCO	72	EM BRANCO	16	136
SIM	15	SIM	11	SIM	2	28
NÃO	5.600	NÃO	8.090	NÃO	1.296	**14.986**
IGNORADO	473	IGNORADO	329	IGNORADO	28	830
**ENCAMINHAMENTO: CENTRO DE REFERÊNCIA DOS DIREITOS HUMANOS**						
EM BRANCO	48	EM BRANCO	75	EM BRANCO	16	139
SIM	5	SIM	4	SIM	X	9

NÃO	5.603	NÃO	8.091	NÃO	1.298	**14.992**
IGNORADO	480	IGNORADO	332	IGNORADO	28	812
**ENCAMINHAMENTO: MINISTÉRIO PÚBLICO**						
EM BRANCO	48	EM BRANCO	75	EM BRANCO	16	139
SIM	4	SIM	9	SIM	X	13
NÃO	5.605	NÃO	8.084	NÃO	1.298	**14.987**
IGNORADO	479	IGNORADO	334	IGNORADO	28	841
**ENCAMINHAMENTO: DELEGACIA DE PROTEÇÃO À CRIANÇA E AO ADOLESCENTE**						
EM BRANCO	48	EM BRANCO	75	EM BRANCO	16	139
SIM	11	SIM	18	SIM	4	33
NÃO	5.606	NÃO	8.079	NÃO	1.294	**14.979**
IGNORADO	471	IGNORADO	330	IGNORADO	28	829
**ENCAMINHAMENTO: DELEGACIA DE ATENDIMENTO À MULHER**						
EM BRANCO	48	EM BRANCO	75	EM BRANCO	16	139
SIM	22	SIM	20	SIM	4	46
NÃO	5.592	NÃO	8.073	NÃO	1.293	**14.958**
IGNORADO	474	IGNORADO	334	IGNORADO	29	837
**ENCAMINHAMENTO: OUTRAS DELEGACIAS**						
EM BRANCO	48	EM BRANCO	73	EM BRANCO	16	137
SIM	76	SIM	128	SIM	17	221
NÃO	5.529	NÃO	7.965	NÃO	1.281	**14.775**
IGNORADO	483	IGNORADO	336	IGNORADO	28	847
**ENCAMINHAMENTO: JUSTIÇA DA INFÂNCIA E JUVDENTUDE**						
EM BRANCO	48	EM BRANCO	75	EM BRANCO	16	139
SIM	9	SIM	29	SIM	4	42
NÃO	5.607	NÃO	8.067	NÃO	1.294	**14.968**
IGNORADO	472	IGNORADO	331	IGNORADO	28	831
**ENCAMINHAMENTO: DEFENSORIA PÚBLICA**						
EM BRANCO	49	EM BRANCO	75	EM BRANCO	16	140
SIM	5	SIM	7	SIM	1	13
NÃO	5.604	NÃO	8.085	NÃO	1.295	**14.984**
IGNORADO	478	IGNORADO	335	IGNORADO	30	843
