# Supplementary material for: Self-harm in the two years of greatest restrictions during the covid-19 pandemic: a cross-sectional study
Source: Rev Bras Enferm. 2025 Jan 10;77(Suppl 2):e20240289. doi: 10.1590/0034-7167-2024-0289 (PMC11726816; doi:10.1590/0034-7167-2024-0289)
Supplement: Translation

# Translation: Violência autoprovocada nos dois anos de maior restrição da pandemia de covid-19: estudo transversal

**DOI:** 10.1590/0034-7167-2024-0289pt

**Authors:** Odilon Vieira Santos, Letícia Aparecida Machado Lisboa da Silva, Meiry Fernanda Pinto Okuno, Paula Hino, José Ramón Martínez-Riera, Hugo Fernandes

**Affiliations:** I Universidade Federal de São Paulo. São Paulo, São Paulo, Brasil; II Universidad de Alicante. Alicante, España

**Keywords:** Violência, Tentativa de Suicídio, Epidemiologia, Sistemas de Informação em Saúde, Vigilância em Saúde Pública

## Abstract

**Objetivo:**

analisar a ocorrência de violência autoprovocada, o perfil sociodemográfico das vítimas e os encaminhamentos nos primeiros 24 meses da pandemia de COVID-19 em São Paulo.

**Método:**

estudo transversal, realizado pelo Sistema de Informação de Agravos de Notificação com dados de violência autoprovocada em São Paulo. O período delineado foi março de 2020 a fevereiro de 2022. Utilizaram-se o *software* R (4.0.2) e teste qui-quadrado.

**Resultados:**

ocorreram 15.946 ocorrências. As vítimas foram mulheres, jovens, brancas, solteiras e heterossexuais. Houve elevada ocorrência em pessoas com transtornos mentais prévios mais de uma vez e sem motivação clara. O meio utilizado foi o envenenamento/intoxicação. Houve número considerável de encaminhamentos à rede de saúde, porém não totalitário.

**Conclusão:**

os anos de maior insegurança em relação à pandemia suscitaram casos de violência autoprovocada com características peculiares. Políticas de saúde ágeis devem ser aplicadas em condições atípicas, como pandemias, especialmente para adolescentes/jovens com transtornos mentais prévios.

## INTRODUÇÃO

A violência autoprovocada, também conhecida como autolesão ou automutilação, refere-se a comportamentos em que indivíduos infligem danos físicos a si mesmos como uma forma de lidar com emoções intensas, angústia emocional, estresse, ou para expressar dor interior de alguma forma tangível. Uma das formas mais sérias de violência autoprovocada é a tentativa de suicídio, na qual a pessoa deliberadamente tenta tirar a própria vida^(1)^.

A tentativa de suicídio é um comportamento extremamente sério que geralmente resulta da interação complexa de diversos fatores, incluindo fatores biológicos, psicológicos, sociais e ambientais. Muitas vezes, as pessoas que tentam suicídio estão lutando contra sentimentos de desesperança, solidão, desamparo e dor emocional intensa. A ideação suicida pode ser influenciada por transtornos mentais, como depressão grave, transtorno bipolar, esquizofrenia, entre outros. Traumas passados, eventos estressantes recentes, problemas financeiros, isolamento social e falta de apoio também podem desempenhar um papel significativo^(1,2)^.

A pandemia de COVID-19 trouxe consigo não apenas desafios de natureza sanitária, mas também impactos profundos na saúde mental das pessoas ao redor do mundo. Nesse contexto, a manifestação de comportamentos de violência autoprovocada e tentativas de suicídio emergiu como uma preocupação significativa. A interação complexa entre os efeitos diretos e indiretos da pandemia contribuiu para o agravamento dos problemas de saúde mental e a expressão desses comportamentos autodestrutivos em muitos indivíduos^(3)^. Uma das principais razões para esse aumento reside no isolamento social imposto pelas medidas de distanciamento social. A ausência de interações sociais presenciais e o suporte emocional resultante desse isolamento prolongado têm alimentado sentimentos de solidão, tristeza e desesperança. Esses sentimentos intensificados levaram pessoas a comportamentos como a automutilação como uma forma de aliviar a dor emocional que enfrentam^(3,4)^.

Estudo norte-americano cita que a crise econômica desencadeada pela pandemia também contribuiu para o estresse financeiro e a incerteza sobre o futuro^(5)^. A perda de empregos, a redução de renda e a insegurança financeira generalizada sobrecarregaram a capacidade de enfrentamento das pessoas, tornando-as mais propensas a problemas de saúde mental e ações autodestrutivas. A incerteza em relação à saúde pessoal e de entes queridos, o medo da doença e da morte também desempenharam um papel na intensificação desses comportamentos.

Pesquisas^(6,7)^ apontaram para o aumento de casos de depressão, ansiedade, pânico, mania e disforia. Além disso, problemas sociais tornaram-se mais evidentes desde o início da pandemia, especialmente em momentos de restrição de atividades comerciais impostas como medida complementar à redução de circulação de pessoas em momentos epidemiológicos de aumento de número de casos e riscos de colapsos no sistema de saúde pública. Assim, somados os fatores elencados, muitas pessoas podem desenvolver condições que lhes afetam tão íntima e fortemente que podem desencadear comportamentos de violência autoprovocada e/ou tentativa de suicídio.

A violência é uma preocupação de saúde pública há anos, integrando a lista de agravos de notificação compulsória desde 2009, incluindo sua forma autoprovocada e a tentativa de suicídio. Essa última trata-se de notificação imediata, ou seja, o notificador deve informar os órgãos de vigilância epidemiológica logo após o atendimento inicial à vítima, para que medidas de controle e assistência especializada possam ser realizadas rapidamente, evitando recidivas do comportamento suicida ou agravamentos do quadro psíquico^(8)^.

Revisão sistemática, que teve como objetivo analisar a incidência de tentativas de suicídio e suas tendências durante a pandemia de COVID-19, apontou a baixa quantidade de estudos epidemiológicos sobre a temática que apontassem com detalhamento as características das ocorrências, as causas e as medidas adotadas pelo setor saúde no atendimento das vítimas^(9)^. Assim, surgiram nos autores os seguintes questionamentos: quais são as características sociodemográficas das vítimas de violência autoprovocada durante a pandemia de COVID-19, as condições de sua ocorrência e quais foram os encaminhamentos feitos pelos profissionais de saúde? Estima-se que os resultados possam contribuir para a implementação de políticas em saúde de prevenção, controle do agravo em situações atípicas, como pandemias e catástrofes.

## OBJETIVO

Analisar a ocorrência de violência autoprovocada, o perfil sociodemográfico das vítimas e os encaminhamentos nos primeiros 24 meses da pandemia de COVID-19 em São Paulo.

## MÉTODOS

### Aspectos éticos

O estudo foi conduzido de acordo com os princípios éticos estabelecidos para pesquisas. Como os dados foram coletados a partir de uma base de dados de domínio público e amplamente acessível, não foi necessário submeter o projeto a um Comitê de Ética em Pesquisa, seguindo as normas internacionais e a Resolução n^o^. 466/12 do Conselho Nacional de Saúde.

### Tipo de estudo

Este estudo possui uma abordagem descritiva e exploratória, com desenho transversal^(10)^, seguindo as diretrizes do instrumento *STrengthening the Reporting of OBservational studies in Epidemiology* (STROBE)^(11)^.

### Coleta de dados

Os dados foram obtidos na cidade de São Paulo, SP. A obtenção de informações foi conduzida através das Fichas de Notificação de Casos Suspeitos ou Confirmados de Violência Interpessoal e Autoprovocada do Sistema de Informação de Agravos de Notificação (SINAN). As informações foram registradas e armazenadas no SINAN por diversos serviços notificadores, abrangendo vigilância epidemiológica, centros hospitalares e ambulatórios. Após a etapa de coleta, as informações foram disponibilizadas pela Secretaria Municipal de Saúde de São Paulo no *software* tabulador TabNet, desenvolvido pelo Departamento de Informática do Sistema Único de Saúde e acessível de forma *online*.

São Paulo foi escolhida em virtude de sua proeminência como a cidade mais populosa do Brasil, aliada à sua marcante diversidade social e cultural. Adicionalmente, os dados de notificação na cidade apresentavam maior facilidade de acesso para consulta pública, por meio do TabNet, em contraste com o sistema nacional. Enquanto as informações da capital paulista estavam disponíveis até 2022, o sistema nacional oferecia dados tabulados apenas até 2020.

### Variáveis estudadas

O estudo analisou variáveis das vítimas, incluindo faixa etária, gênero, raça/cor da pele, presença de gestação, escolaridade, coordenadoria de saúde de residência da vítima, situação conjugal, orientação sexual, identidade de gênero, presença de transtorno ou deficiência e seu tipo. Em relação às ocorrências, foram analisados mês, local, se ocorreu mais de uma vez, motivação, meio de agressão, suspeita do uso de álcool e os encaminhamentos feitos pelos profissionais após o atendimento das vítimas.

### Critérios de elegibilidade

Foram utilizadas as notificações de violência autoprovocada, feitas em estabelecimentos de saúde, tanto públicos quanto privados, localizados em São Paulo, SP. Excluíram-se os casos de violência interpessoal e as duplicidades de notificações. Em razão da uniformidade da ficha de notificação para o espectro de casos suspeitos e confirmados de violência interpessoal, uma segregação entre essas categorias revelou-se inviável. Ademais, cumpre observar que o programa TabNet não concede a distinção entre os casos confirmados e suspeitos. Nesse contexto, optou-se por contemplar a integralidade de ocorrências de violência autoprovocada, quer fossem elas tidas como suspeitas ou já confirmadas no momento da notificação.

### Período de análise

A coleta de dados abrangeu o período de março de 2020 a fevereiro de 2022, totalizando 24 meses. A escolha desse intervalo justifica-se pelo fato de que a declaração de pandemia pela Organização Mundial da Saúde (OMS) ocorreu em março de 2020, e as medidas de distanciamento social começaram a ser implementadas em São Paulo dias depois. A coleta das informações foi realizada entre dezembro de 2022 e fevereiro de 2023. Para a análise dos dados, o programa *Microsoft Excel* 365^®^ foi utilizado.

### Tratamento e análise dos dados

A análise estatística univariada foi conduzida utilizando o *software* R, versão 4.0.2. Dado que o conjunto de dados é composto por variáveis categóricas, realizou-se uma análise descritiva por meio da contagem de frequências absolutas e percentuais. Para examinar as associações entre a violência e outras variáveis, como motivação da violência, meio de agressão, local, gênero do agressor, encaminhamento, faixa etária, raça, educação, estado civil e deficiência, foi empregado o teste qui-quadrado. O nível de significância adotado foi de 5% (α = 0,05).

## RESULTADOS

No período delineado, houve 15.946 notificações de violência autoprovocada em São Paulo, SP. Foram encontrados valores estatisticamente significativos na maioria das variáveis sociodemográficas, com exceção de “gestação” (p=0,460) e “deficiência física” (p=0,086), conforme mostra a Tabela 1.

**Tabela 1:** Notificações de violência autoprovocada segundo características sociodemográficas das vítimas. São Paulo, SP, Brasil, 2023

Variável	n	%	Valor de p^*^
Faixa etária (anos)			<0,001
0 - 4	177	1,1	
5 - 9	68	0,4	
10 - 14	1605	10	
15 - 19	3677	23	
20 - 24	2996	18,8	
25 - 29	1963	12,3	
30 - 34	1444	9	
35 - 39	1230	7,8	
40 - 44	994	6,2	
45 - 49	661	4,2	
50 - 54	453	2,8	
55 - 59	256	1,7	
60 - 64	179	1,1	
65 - 69	112	0,7	
70 - 74	59	0,4	
75 e mais	73	0,5	
Sexo			<0,001
Feminino	11141	69,9	
Masculino	4804	30,1	
Raça/cor			0,037
Ignorado/em branco	1116	7	
Branca	7702	48,3	
Preta	1479	9,3	
Amarela	121	0,7	
Parda	5481	34,4	
Indígena	48	0,3	
Gestação			0,460
Ignorado/em branco	2339	14,7	
Sim	271	1,7	
Não	7242	45,4	
Não se aplica	6095	38,3	
1º trimestre	99	0,6	
2º trimestre	97	0,6	
3º trimestre	51	0,3	
Idade gestacional ignorada	24	0,1	
Escolaridade			0,009
Ignorado/em branco	6132	38,5	
Analfabeto	53	0,3	
1ª a 4ª série incompleta do ensino fundamental	296	1,9	
5ª a 8ª série incompleta do ensino fundamental	231	1,4	
Ensino fundamental completo	1723	10,8	
Ensino médio incompleto	861	5,4	
Ensino médio completo	2230	14,0	
Ensino superior incompleto	3027	19,0	
Ensino superior completo	639	4,0	
Não se aplica	578	3,6	
Coordenadoria de saúde de residência (zona)			<0,001
Oeste	973	6,1	
Leste	2504	15,7	
Norte	3095	19,4	
Sudeste	2776	17,4	
Sul	4713	29,6	
Centro	381	2,4	
Em branco	1523	9,6	
Não informado	8	0,1	
Situação conjugal			<0,001
Em branco	204	1,3	
Solteiro(a)	7684	48,2	
Casado/união consensual	2609	16,4	
Viúvo(a)	117	0,7	
Separado(a)	575	3,6	
Não se aplica	1104	6,9	
Ignorado	3680	23,1	
Orientação sexual			<0,001
Heterossexual	8006	50,2	
Homossexual	597	3,7	
Bissexual	262	1,6	
Não se aplica	1111	7,0	
Ignorado	5997	37,6	
Identidade de gênero			<0,001
Travesti	35	0,2	
Mulher transexual	182	1,1	
Homem transexual	135	0,8	
Não se aplica	9205	57,7	
Ignorado	6416	40,2	
Presença de deficiência ou transtorno			<0,001
Em branco	62	0,4	
Sim	4644	29,1	
Não	7862	49,3	
Ignorado	3405	21,4	
Deficiência física			0,086
Em branco	162	1,0	
Sim	100	0,6	
Não	4311	27,0	
Não se aplica	11264	70,6	
Ignorado	136	0,9	
Deficiência intelectual			<0,001
Em branco	158	1,0	
Sim	307	1,9	
Não	4109	25,8	
Não se aplica	11264	70,6	
Ignorado	135	0,8	
Transtorno mental			<0,001
Em branco	124	0,8	
Sim	2036	12,8	
Não	2454	15,4	
Não se aplica	11264	70,6	
Ignorado	95	0,6	
Transtorno de comportamento			<0,001
Em branco	121	0,8	
Sim	2260	14,2	
Não	2242	14,1	
Não se aplica	11264	70,6	
Ignorado	86	0,5	

* *Teste Qui-quadrado.*

Evidencia-se maior frequência de notificações de violência autoprovocada sofrida por pessoas com faixa etária entre 15 e 19 anos (23%; n= 3.677), seguida por pessoas entre 20 e 24 anos (18,8%; n= 2.996), do sexo feminino (69,9%; n=11.151), de cor branca (48,3%; n= 7.702), solteiras (48,2%; n=7.684), heterossexuais (50,2%; n= 8.006) e residentes na região Sul da coordenadoria de saúde (29,6%; n= 4.713). Em relação à escolaridade, notou-se elevado número de registros classificados como “ignorado/em branco” (38,5%; n= 6.132).

Quanto à presença de deficiência ou transtorno, a maioria não possuía essas condições (49,3%; n=7.862). Porém, em casos afirmativos, os transtornos de comportamento foram os mais prevalentes (14,2%; n=2.260), seguidos dos transtornos mentais (12,8%; n=2.036).

As principais características em relação à ocorrência da violência autoprovocada, como local da ocorrência, se ocorreu outras vezes, motivação e meio de agressão, são apresentadas na Tabela 2.

**Tabela 2:** Notificações de violência autoprovocada segundo características da ocorrência. São Paulo, SP, Brasil, 2023

Variável	n	%	Valor de p^*^
Local da ocorrência			0,033
Em branco	4	0,0	
Residência	12197	76,5	
Habitação coletiva	108	0,7	
Escola	53	0,3	
Local de prática esportiva	5	0,0	
Bar ou similar	20	0,1	
Via pública	782	4,9	
Comércio/serviços	122	0,8	
Indústria/construção	5	0,0	
Outros	429	2,7	
Ignorado	2255	14,1	
Ocorreu outras vezes			
Em branco	192	1,2	0,002
Sim	7182	45,0	
Não	4816	30,2	
Ignorado	3790	23,8	
Motivação			
Sexismo	115	0,7	<0,001
Homofobia/transfobia	77	0,5	
Racismo	10	0,1	
Intolerância religiosa	5	0,0	
Xenofobia	5	0,0	
Conflito geracional	911	5,7	
Situação de rua	43	0,3	
Deficiência	117	0,7	
Outros	5405	33,9	
Não se aplica	3105	19,5	
Ignorado	6187	38,8	
Meio de agressão			
Força corporal	278	1,7	<0,001
Enforcamento	578	3,6	
Objeto contundente	194	1,2	
Objeto perfurocortante	3408	21,4	
Substância ou objeto quente	107	0,7	
Envenenamento ou intoxicação	9733	61,0	
Arma de fogo	68	0,4	
Ameaça	309	1,9	
Outro meio	1626	10,2	
Suspeita do uso de álcool			
Em branco	111	0,7	0,004
Sim	2360	14,8	
Não	8665	54,3	
Ignorado	4844	30,4	

* *Teste Qui-quadrado.*

A Tabela 2 evidencia que o local mais comum da violência autoprovocada foi a residência da vítima (76,5%; n=12197) e que a agressão ocorreu com frequência superior a uma vez (45%; n= 7182). Já a motivação principal da autoagressão não fica clara, tendo em vista que a maioria das notificações teve o campo “ignorado” como o mais preenchido (38,8%; n= 6.187), seguido por “outros” (33,9%; n= 5.405).

O meio de agressão mais empregado foi o envenenamento ou intoxicação (61%; n= 9.733), e não esteve associado ao consumo de álcool na maioria das ocorrências (54%; n= 8.665).

A síntese dos encaminhamentos realizados pelos profissionais após o atendimento às vítimas é apresentada na Tabela 3.

**Tabela 3:** Encaminhamentos realizados pelos profissionais após o atendimento da vítima de violência autoprovocada. São Paulo, SP, Brasil, 2023

Encaminhamentos	n	%	Valor de p^*^
Rede de Atenção à Saúde	13558	76,5	<0,001
Rede de Assistência Social	910	5,1	
Rede de Educação	55	0,3	
Rede de Atendimento à Mulher	519	2,9	
Conselho Tutelar	2276	12,8	
Conselho do Idoso	28	0,2	
Centro de Referência em Direitos Humanos	9	0,1	
Ministério Público	13	0,1	
Delegacia de Proteção à Criança e ao Adolescente	33	0,2	
Delegacia de Atendimento à Mulher	46	0,3	
Outras delegacias	221	1,2	
Justiça da Infância e Juventude	42	0,2	
Defensoria Pública	13	0,1	
Total	17723	100	

* *Teste Qui-quadrado.*

Nota-se que os profissionais encaminharam as vítimas de violência autoprovocada, principalmente, para serviços que compõem a Rede de Atenção à Saúde (76,5%; n=13558). Destaca-se que, neste campo, os profissionais podiam sinalizar mais de um tipo de encaminhamento. Assim, o valor total (n=17723) é superior ao número de casos atendidos no período delineado (n= 15946).

## DISCUSSÃO

A violência tem consequências profundas para a saúde física e mental das pessoas que a vivenciam, tendo impacto no desenvolvimento e bem-estar psicossociais. O presente estudo identificou que jovens entre 15 e 24 anos foram as principais vítimas de violência autoprovocada e tentativa de suicídio durante a pandemia. Segundo a OMS^(12)^, o suicídio é a quarta principal causa de morte entre jovens nessa faixa etária, sendo que a taxa de suicídio entre adolescentes aumentou em muitos países nas últimas décadas. A mortalidade entre adolescentes e jovens adultos compromete o futuro, apontando para a negligência de governos e sociedades em garantir a vida plena e com qualidade nessa faixa etária.

Estudo brasileiro, realizado no início da pandemia de COVID-19, com registros assistenciais de 130 pacientes atendidos após ideação ou tentativa de suicídio na emergência psiquiátrica de um serviço de saúde mental, identificou que houve elevada incidência de comportamento suicida entre adolescentes com idade a partir de 13 anos^(13)^. Outras pesquisas apontam que os fatores associados à violência autoprovocada por adolescentes no contexto pandêmico estiveram associados a aspectos ambientais, emocionais, genéticos e fisiológicos, além de outras condições, como ansiedade e abuso de substâncias ilícitas^(1,5)^.

Já em relação ao gênero, homens e mulheres podem ser vítimas de violência autoprovocada ao longo de diferentes momentos do ciclo vital. Porém, as mulheres sofrem com maior frequência de transtornos mentais e condições socioambientais que as colocam em maior condição de vulnerabilidade de tentativa de suicídio. Estudo canadense sobre a temática durante a pandemia de COVID-19 apontou que as mulheres apresentaram maior frequência de ideação suicida e que aspectos relacionados ao isolamento social, aumento de desemprego, falta de rede de apoio, insegurança quanto ao futuro próximo e aumento de demandas domésticas foram fatores propulsores desse comportamento^(14)^.

A vulnerabilidade é ainda maior quando considerada a intersecção entre gênero, pobreza e raça/cor, fazendo com que mulheres pardas e pretas tivessem maior ocorrência de ideação suicida. Todavia, esse aspecto não foi evidenciado neste estudo, tendo em vista que a maioria das vítimas se autodeclarou como branca. Vale destacar que a autodeclaração de cor de pele no Brasil é um assunto delicado, em virtude do racismo estrutural encontrado no país, fazendo com que pessoas negras muitas vezes não se reconheçam como tal^(15)^. Já a condição de pobreza não foi investigada neste estudo, mas a alta ocorrência do agravo na região Sul do município, onde estão presentes grandes áreas de pobreza e intensa desigualdade social, pode sugerir tal intersecção.

Quanto às características referentes à situação conjugal, há evidência na literatura que descreve a prevalência das tentativas de suicídio e suicídio em pessoas solteiras. Dados de pesquisa retrospectiva quantitativa que analisou o perfil epidemiológico das tentativas e mortes por suicídio no Brasil entre 2009 e 2018 revelou que o estado civil da maior parte das vítimas em todas as regiões do território brasileiro era solteiro^(16)^. Igualmente, nas análises de pesquisadores da Universidade de Brasília, foram constatadas maiores porcentagens de óbitos por suicídio no Distrito Federal em solteiros, viúvos e separados judicialmente, em comparação aos indivíduos que mantêm uma união estável^(17)^.

Outro resultado encontrado refere-se ao comportamento e prática suicida em indivíduos declarados heterossexuais. Os dados encontrados no presente trabalho divergem de estudo nacional que aponta maior vulnerabilidade e ocorrência em indivíduos não heterossexuais, justificada pela influência de fatores discriminatórios vivenciados por homossexuais e bissexuais^(18)^. A literatura internacional também aponta maior prevalência do comportamento suicida em pessoas não heterossexuais^(19)^. Dessa forma, os achados desta pesquisa revelam que, na pandemia de COVID-19, as discriminações associadas à homossexualidade ou bissexualidade não foram motivadoras principais para a autoagressão.

Elevada frequência de resultados “outros”, “em branco” ou “ignorado” em campos, como a escolaridade das vítimas, pode indicar falhas no treinamento dos notificadores, necessitando de aumento da conscientização de que a notificação compulsória é uma importante ferramenta não apenas para o acompanhamento e investigação dos casos de violência, mas também para servir de instrumento fundamental para a tomada de decisões em saúde, como a implementação de políticas públicas^(20)^, fato encontrado nesta pesquisa.

Pessoas portadoras de transtornos de comportamento e transtornos mentais prévios apresentaram maior frequência de violência autoprovocada. Nesses grupos, incluem-se transtornos de personalidade antissocial, *borderline*, transtorno de personalidade histriônica, depressão, ansiedade, transtorno afetivo bipolar, esquizofrenia, entre outras condições. Pesquisadores internacionais citam que houve uma íntima relação entre transtornos mentais e de comportamento com a ideação suicida durante a pandemia, principalmente de adolescentes e jovens adultos^(21)^. Todavia, ainda não está claro que em todos os casos tais transtornos eram previamente existentes, latentes ou se desenvolvidos pelas condições de isolamento social e medo ocasionados na pandemia de COVID-19.

A ocorrência de violência autoprovocada e tentativas de suicídio foi mais comum nas moradias das vítimas, possivelmente em virtude das restrições da pandemia e também porque trata-se de uma violência íntima, que não necessita de espaços públicos para sua ocorrência. Um achado relevante foi o agravo ter ocorrido mais de uma vez em cerca de 45% das notificações. Repetidas autoagressões expressam um sinal de gravidade do estado mental das vítimas, necessitando de rápidas e assertivas intervenções, tendo em vista a alta probabilidade de êxito do suicídio, em virtude do aperfeiçoamento da prática que outrora falhou^(21,22)^. Por ter causas variáveis, sua motivação nem sempre é nítida ou pode ser expressa pelas vítimas de forma clara, fazendo com que os profissionais de saúde que as atendam não identifiquem campos adequados para preencher na ficha de notificação compulsória^(8,20)^.

No que se refere aos meios de agressão, o envenenamento ou intoxicação foi o mais frequente. Contudo, a literatura mostra uma variação nos métodos mais utilizados tanto no cenário nacional quanto internacional^(21-23)^. Dentre os métodos frequentemente empregues para a prática suicida, uma revisão da literatura de caráter epidemiológico mostrou que houve predomínio de mortes autoprovocadas por enforcamento, abrangendo 47% dos casos, seguido por armas de fogo (19%) e envenenamento (14%)^(23)^. Já no panorama internacional, o mesmo estudo apontou a utilização de envenenamento por pesticidas e medicamentos em países do continente asiático, como a China e o Sri Lanka. Ressalta-se que existem fatores atrelados ao contexto socioeconômico e cultural em que a pessoa está inserida que influenciam diretamente a escolha do método. Sendo assim, as formas mais utilizadas podem variar muito.

Da mesma forma, pesquisadores brasileiros descreveram, em estudo transversal, os casos de violência autoprovocada e intoxicação exógena ocorridos no Rio Grande do Sul no período de 2013 a 2017^(24)^. Em conformidade com o achado da análise, foi encontrado na amostra que a maior parte dos casos de violência autoprovocada foram causados por envenenamento ou intoxicação exógena, representando 39,4% das notificações do território gaúcho. Em seguida, ficaram as mortes provocadas por enforcamento, uso de objetos perfurocortantes e armas de fogo, respectivamente.

Por fim, o elevado número de encaminhamentos à Rede de Atenção à Saúde é justificado pela necessidade de atenção especializada em saúde mental requerida nessas situações, além de sinalizar alinhamento com as recomendações da linha de cuidado à pessoa em situação de violência da cidade de São Paulo. Todavia, nota-se que não ocorreu o encaminhamento na totalidade dos casos aos serviços de saúde especializados em saúde mental, como psiquiatras e/ou psicólogos, como recomenda a linha de cuidados supracitada^(25)^. O não encaminhamento rápido a profissionais de saúde especializados pode colocar em risco a integridade das vítimas e aumentar as chances de recidivas^(22,25)^.

### Limitações do estudo

Uma das principais limitações do estudo encontra-se na regionalidade dos fatos, tendo em vista que, por se tratar da maior metrópole da América Latina, o perfil das vítimas e as características da violência perpetrada contra si podem ser diferentes de cidades menores. Além disso, nesta cidade, todas as Unidades Básicas de Saúde possuem Núcleos de Prevenção de Violência, que são compostos por profissionais de saúde e/ou assistência social treinados na abordagem de atendimento às vítimas de violência, seja ela interpessoal ou autoprovocada. Em regiões onde não existam esses núcleos ou linhas de cuidado especializadas, talvez possam existir prejuízos nas notificações e no atendimento das vítimas.

### Contribuições para a área da enfermagem e ciência

Apesar das limitações expostas, o estudo contribui com o avanço da ciência, à medida que explora o ineditismo da temática e apresenta um perfil sociodemográfico e as principais características da violência autoprovocada em uma situação atípica, como a pandemia de COVID-19, e suscita a necessidade de reflexões sobre estratégias futuras para a redução desse agravo em situações similares, incluindo a prevenção em populações altamente vulneráveis, como mulheres adolescentes e jovens adultas.

## CONCLUSÕES

A violência autoprovocada foi elevada durante a pandemia de COVID-19, tendo como principais vítimas mulheres adolescentes e jovens adultas, brancas, solteiras, heterossexuais, residentes na região com maior desigualdade social da cidade. Aquelas com transtornos mentais ou de comportamento tiveram frequência maior do agravo, cabendo à busca de tratamentos e assistências que levem em consideração a vulnerabilidade à autoagressão. Infelizmente, a violência ocorreu mais de uma vez, elevando as chances de êxito na tentativa de suicídio e mostrando potenciais falhas na resolução dos problemas.

O envenenamento ou intoxicação foi o meio mais adotado pelas vítimas, cuja motivação para a ocorrência foi classificada como ignorada. A Rede de Atenção à Saúde foi o principal encaminhamento realizado após o atendimento, e aponta o seguimento das recomendações da linha de cuidado à pessoa em situação de violência.

A pandemia de COVID-19 causou danos inumeráveis à sociedade, com prováveis consequências mentais e comportamentais. Esta pesquisa permitiu dar luz à temática da violência autoprovocada em situações atípicas, como a pandemia. No entanto, é importante destacar que os campos da saúde mental e da vigilância em saúde continuam a se desenvolver em busca de estratégias de redução dos riscos, como notificações compulsórias mais detalhadas e precisas, e pela abordagem desse agravo, de forma holística, que leve em consideração os múltiplos fatores que o influenciam.

## Data Availability

https://osf.io/u7ek8/
